# High remnant cholesterol level is relevant to diabetic retinopathy in type 2 diabetes mellitus

**DOI:** 10.1186/s12944-021-01621-7

**Published:** 2022-01-20

**Authors:** Yongyan Shan, Qian Wang, Yitong Zhang, Xuewei Tong, Shengdan Pu, Yuxin Xu, Xinyuan Gao

**Affiliations:** Endocrinology department, The First Affiliated Hospital (FAH)for Harbin Medical University(HMU), Harbin, 150001 People’s Republic of China

**Keywords:** Diabetic retinopathy, Remnant cholesterol, Nomogram, NLR, DME, TyG-index, Type 2 diabetes mellitus

## Abstract

**Background:**

Diabetic retinopathy (DR) is the primary oculopathy causing blindness in diabetic patients. Currently, there is increasing interest in the role of lipids in the development of diabetic retinopathy, but it remains controversial. Remnant cholesterol (RC) is an inexpensive and easily measurable lipid parameter; however, the relationship between RC and DR in type 2 diabetes mellitus (T2DM) has not been elucidated. This research investigates the relevance between RC levels and DR severity while building a risk prediction model about DR.

**Methods:**

In this single-centre retrospective cross-sectional study. Each hospitalised T2DM patient had no oral lipid-lowering drugs in the past three months, and coronary angiography showed epicardial coronary artery stenosis of less than 50% and completed seven-field stereo photographs, fluorescein fundus angiography, and optical coherence tomography detection. The RC value is calculated according to the internationally recognised formula. Binary logistic regression was used to correct confounding factors, and the receiver operating characteristic (ROC) analysis was used to identify risk factors and assess the nomogram’s diagnostic efficiency.

**Results:**

A total of 456 T2DM patients were included in the study. The RC levels in the DR team was higher [0.74 (0.60–1.12) mmo/l vs 0.54 (0.31–0.83) mmol/l *P* < 0.001] in the non-DR team. After adjusting for confounding elements, RC levels are still associated with DR risk (OR = 5.623 95%CI: 2.996–10.556 *P* < 0.001). The ratio of DR in every stage (except mild non-proliferative diabetic retinopathy) and DME in the high RC level team were further increased compared to the low-level team (all *P* < 0.001). After ROC analysis, the overall risk of DR was predicted by a nomogram constructed for RC, diabetes duration, and the neutrophil-lymphocyte ratio as 0.758 (95%CI 0.714–0.802 *P* < 0.001).

**Conclusions:**

High RC levels may be a potential risk factor for diabetic retinopathy, and the nomogram does better predict DR. Despite these essential findings, the limitation of this study is that it is single-centred and small sample size analysis.

**Supplementary Information:**

The online version contains supplementary material available at 10.1186/s12944-021-01621-7.

## Introduction

As the worldwide prevalence of diabetes mellitus increases, 463 million adults were diagnosed with diabetes in 2019, and according to this trend, there will be 700 million diabetes worldwide by 2045 [1]. Diabetic retinopathy (DR) is a prevalent neurovascular complication of diabetes mellitus and is the leading cause of blindness in the working-age generation [2]. Although glycemic levels, diabetes duration, microalbuminuria, and blood pressure levels are known risk elements for the development and advance of DR, current studies report that there may be other, as yet unknown, risk factors for the disease [3]. The association of dyslipidemia with DR has been thoroughly investigated. However, in many extensive studies, controversy still exists regarding the exact role of dyslipidemia in DR [4, 5]. Perhaps, traditional lipid levels alone are inadequate, although a recent study shows that novel serum biomarkers of lipid profiles and inflammatory reactions are potentially related to DR [6].

Remnant cholesterol (RC) is the cholesterol content of triglyceride-rich lipoproteins, which is IDL and VLDL in the fasting state and the non-fasting state chylomicron remnants [7]. Current studies suggest that RC is derived from two pathways, the endogenous pathway is VLDL produced by the liver, and exogenously, chylomicron is made within the intestine. Triglycerides and chylomicrons in VLDL enter the bloodstream and are hydrolysed by lipoprotein lipase (LPL) at high speed, continuously producing smaller, denser residual lipoprotein particles. RC level assessment is inexpensive and convenient, perhaps providing valuable data for clinical management, and can be easily calculated using an established formula [8]. Recent studies have confirmed that RC positively affects a strong association with cardiovascular endpoints [9, 10]. Whether remnant cholesterol also contributes to the development of microvascular disease in individuals with diabetes is an interesting question. Up to now, the relationship between RC and DR in T2DM patients has not been researched. The present research explored the connection between RC levels and DR in Chinese T2DM patients.

## Methods

### Study design and participants

This retrospective observational cross-sectional study was undertaken in the First Affiliated Hospital of Harbin Medical University. According to the Declaration of Helsinki, the research was ratified by the Ethics Committee of the FAH in HMU. Consecutively selected hospitalised T2DM patients in the endocrinology and cardiology departments in the FAH for HMU between November 2018 and October 2021 were enrolled according to the following criteria. Inclusion criteria are: (1) the diagnosis of T2DM was per 1999 WHO diagnostic criteria [11]; (2) no gender restrictions on participants over 18 years of age; (3) coronary angiography showed epicardial coronary artery stenosis of less than 50%; (4) completed seven-field stereo photographs, fluorescein fundus angiography (FFA), and Optical Coherence Tomography (OCT) detection, and (5) blood lipid and other biochemical indicators were collected on the second day of admission, and the content was complete. Exclusion criteria are: (1) had taken fenofibrate or other oral lipid-lowering drugs during the last three months; (2) definite cerebral infarction or cerebral revascularisation; (3) peripheral arterial occlusive disease; (4) any uncontrolled systemic disease other than sub-optimally controlled diabetes mellitus; (5) other ophthalmic diseases affect DR diagnosis, such as severe cataracts, and (6) any confirmed malignancies.

### Clinical data collection and laboratory analysis

Demographic and physical data taken from the medical records included age, gender, diabetes duration, smoking, medical history, hypertension history, diabetic therapy, blood pressure, body mass index (BMI), and the diagnosis of diabetic peripheral neuropathy (DPN). Venous blood samples were collected after 1-night of fasting to determine Glycosylated Hemoglobin (HbA1c) by Ion Exchange High-Performance Liquid Chromatography (HPLC). The fasting blood glucose (FBG), total cholesterol (TC), triglycerides (TG), uric acid (UA), and creatinine (Cr) levels were determined using enzymatic methods. Fasting C-peptide was assessed using radioimmunoassay. Low-density lipoprotein cholesterol (LDL-C) and high-density lipoprotein cholesterol (HDL-C) levels were determined by homogeneous assays. An automated haematology analyser took white cell counts and differentials (model XT 2000i; Sysmex, Kobe, Japan). Microalbuminuria levels measurements were attained using a 24-h urine collection. According to the recommendation of dyslipidaemia guidelines [12], RC was computed as TC minus LDL-C and HDL-C; where eGFR = 141*min(Scr/κ,1)^α^ × max(SCr/k,1)^1.209^ × 0.993^age^ × gender×ethnic (For females, use the following values: gender = 1.018; α = 0.329; κ = 0.7; For males, use the following values: gender = 1; α = 0.411; κ = 0.9) [13]. The TyG index was calculated using [14]: Ln [fasting triglycerides (mg/dl) × fasting glucose (mg/dl)] / 2. NLR values were obtained by dividing the neutrophil count by the lymphocyte count [15].

### Assessment of DR and DME

Detection of the fundus was achieved by performing seven-field stereo photographs and FFA. The International Clinical Diabetic Retinopathy Disease Severity Scale was used [16] to grade DR. DR was divided into five stages of non-DR, non-proliferative diabetic retinopathy (mild, moderate, severe), and proliferative diabetic retinopathy (PDR). OCT was used to measure the thickness of macular fovea in eyes with macular oedema and determine the severity of the diabetic macular oedema using the International Clinical Diabetic Macular Oedema Disease Severity Scale.

### Statistical analysis

Groups were divided according to the presence or absence of diabetic retinopathy. The statistical software SPSS 26.0 and “R language” statistical packages were used for the statistical analyses. The data’s normalcy was assessed using the Shapiro-Wilk test. The continuous normality variables were introduced as the mean ± SD and evaluated using the Student’s t-test. The skewed continuous variables were shown as the median (interquartile range, IQR) and were assessed using the Mann-Whitney U test. The Chi-square test was used to compare categorical variables. Spearman correlation and partial correlation analysis were used to analyse the connection between RC and DR stages. Spearman correlation analysis also examined the relationship between RC and other variables. The *P*-value of *P* < 0.05 was defined as statistically significant.

The univariate binary logistic regression analyses were used to analyse the presence of DR as the dependent variable and risk elements of DR as independent variables. Those variables which were statistically significant in the univariate analysis but did not mutually interfere were included in the multiple binary regression analysis models. Outcomes of these regression analyses were assessed using ORs, 95%CI, and *p*-values. Hosmer-Lemeshow goodness of fit implementation was used in the optimal model in multivariate regression analysis to select variables to develop a nomogram of the prediction of DR occurrence. The receiver operating characteristic (ROC) analysis was used to identify risk factors and assess the nomogram’s diagnostic efficiency.

The RC level was divided into three groups, according to tertile. The composition ratio of DR in each group, composition ratio at each DR stage, and DME composition ratio were analysed. Chi-square implementation was used to compare teams; *P* < 0.05 was deemed statistically significant.

## Results

The cohort of 456 subjects included 217 participants with DR and 239 without DR. The average age of the subjects was 53.54 ± 12.13, and the mean diabetes duration was 9.73 ± 6.97 years. The number and percentage of mild-NPDR, moderate-NPDR, severe-NPDR, PDR and DME in DR were 57 (26.3%), 67 (30.9%), 57 (26.3%), 36 (16.6%) and 55 (25.3%), respectively. Age, diabetes duration, SBP, antihypertensive drugs, TC, RC, mAlb, TyG-index, neutrophils, and NLR in the DR team were dramatically different from those in the non-DR team (all *P* < 0.05). Meanwhile, the eGFR level was lower (*P* = 0.013), with no significant differences in other variables (all *P* > 0.05, please see Table 1).

**Table 1:** Characteristics of the non-DR group and DR group

Variables	All (***n*** = 456)	Non-DR (***n*** = 239)	DR (***n*** = 217)	***P***-value
Age (years)	53.54 ± 12.13 / 55 (44–63)	53 (40–62)	57 (47–64)	0.009**
Male gender, n (%)	292 (64)	161 (65.2)	131 (62.7)	0.579
Diabetes duration (years)	9.73 ± 6.97 / 9 (4–15)	7 (3–12)	11 (6–16)	0.001**
Hypertension, n (%)	232 (46.4)	105 (42.5)	127 (50.2)	0.085
Current smoking, n (%)	150 (32.9)	89 (36)	61 (29.2)	0.121
DNP, n (%)	175 (38.5)	79 (33.2)	96 (44.2)	0.027
BMI (kg/m^2^)	25.6 (23.92–27.95)	25.39 (23.84–27.39)	25.6 (23.98–28.09)	0.929
SBP (mmHg)	140 (126–150)	137 (123–150)	140 (130–154)	0.007*
DBP (mmHg)	80 (74–90)	80 (72–88)	81 (75–90)	0.067
FBG (mmol/L)	8.44 (6.88–10.61)	8.2 (6.79–10.54)	8.59 (7.04–10.71)	0.163
HbA1c (%)	8.4 (7.2–9.6)	8.2 (7.1–9.5)	8.5 (7.6–9.8)	0.076
HbA1c (mmol/mol)	68 (55–81)	66 (54–80)	69 (60–84)	0.057
Fasting C-Peptide (ng/ml)	1.5 (1.0–2.2)	1.55 (1.0–2.3)	1.5 (1.0–2.15)	0.426
Neutrophil (× 10^9^/L)	3.71 (3.05–4.45)	3.48 (2.96–4.41)	3.85 (3.17–4.5)	0.01*
Lymphocyte (×10^9^/L)	2.22 (1.78–2.64)	2.26 (1.87–2.69)	2.17 (1.73–2.56)	0.053
Monocyte (×10^9^/L)	0.38 (0.31–0.45)	0.38 (0.3–0.45)	0.38 (0.31–0.44)	0.31
TC (mmol/L)	5.02 (4.42–5.74)	4.96 (4.24–5.58)	5.13 (4.58–5.96)	0.000**
TG (mmol/L)	2.18 (1.4–3.43)	2.12 (1.43–3.2)	2.31 (1.35–3.59)	0.286
HDL-C (mmol/L)	1.15 (0.99–1.3)	1.11 (0.99–1.28)	1.16 (1.00–1.325)	0.496
LDL-C (mmol/L)	3.09 (2.66–3.58)	3.14 (2.65–3.59)	3.05 (2.66–3.55)	0.822
APOA (g/L)	1.26 (1.13–1.39)	1.25 (1.14–1.39)	1.21 (1.11–1.40)	0.981
APOB (g/L)	1.03 (0.87–1.21)	1.035 (0.84–1.2)	1.03 (0.9–1.22)	0.2
RC (mmol/L)	0.65 (0.45–0.99)	0.54 (0.31–0.83)	0.74 (0.60–1.12)	0.000**
TyG-index	9.62 ± 0.78	9.52 ± 0.73	9.72 ± 0.82	0.006*
NLR	1.64 (1.36–2.03)	1.60 (1.30–1.93)	1.69 (1.41–2.26)	0.000**
Uric Acid (μmol/L)	329.3 (280.4–393.8)	326.3 (276.6–395)	330.8 (286.05–393.7)	0.644
Creatinine (μmol/L)	61.3 (50.6–70.5)	60.5 (50.45–70.13)	62.3 (51.1–72.4)	0.384
eGFR (ml/min/1.73m^2^)	108.6 (97.68–121.03)	110.96 (98.24–123.10)	106.27 (96–117.95)	0.013
mAlb (mg/24 h)	20.31 (19.76–33.63)	19.95 (19.57–23.56)	20.71 (19.95–56.53)	0.000**
Metformin, n (%)	277 (60.7)	149 (60.3)	128 (61.2)	0.841
DPP-IV inhibitor, n (%)	189 (41.4)	98 (39.7)	91 (43.5)	0.404
Acarbose, n (%)	68 (15.3)	38 (15.9)	30 (14.6)	0.712
Insulin, n (%)	139 (30.5)	72 (29.1)	67 (32.1)	0.502
Anitihypertensive drugs, n (%)	159 (34.9)	76 (30.8)	83 (39.7)	0.046*

Abbreviations: *DR* diabetic retinopathy, *DPN* diabetic peripheral neuropathy, *BMI* body mass index, *SBP* systolic blood pressure, *DBP* diastolic blood pressure, *FBG* Fasting blood glucose, *APOA* apolipoprotein A, *APOB* apolipoprotein B, *TC* total cholesterol, *TG* triglyceride, *HDL-C* high-density lipoprotein cholesterol, *LDL-C* low-density lipoprotein cholesterol, *RC* remnant cholesterol, *eGFR* estimated glomerular filtration rate, *HbA1c* glycosylated hemoglobin, *TyG-index* triglyceride glucose index, *NLR* neutrophil to lymphocyte ratio, *DR* diabetic retinopathy, *DPP-IV* dipeptidyl peptidase-IV, *mAlb* microalbuminuria

Notes: Data are expressed as mean ± SD, percentages, or as medians (IQR); *p-*values were compared by independent t-test, Mann-Whitney U test, or Chi-square test as appropriate.*P < 0.05, **P < 0. 001

### Correlation between RC and other variables in the lipid profiles with stages of DR

The correlation between RC and other variables in the lipid profiles (including TC, TG, LDL-C, HDL-C, ApoA, and ApoB) with the DR stages was assessed using Spearman correlation analysis. Table 2 shows that only RC and TC were correlated with the DR stages (r = 0.360 *P* < 0.001) and (r = 0.142 *P* = 0.002), respectively. Even after controlling TC by partial correlation analysis, RC (r = 0.298 *P* < 0.001) was still correlated with DR stages. In the univariate analysis, the risk factors of DR, age, diabetes duration, DNP, oral antihypertensive drugs, systolic blood pressure, TC, RC, mAlb, TyG, and NLR were statistically significant. After adjusting confounding factors using binary logistic regression analysis, RC (OR = 5.623 95%CI: 2.996–10.556 *P* < 0.001) was still a risk factor for DR, as shown in Tables 3 and S1. Moreover, the variables of NLR(OR = 1.742 95% CI: 1.218–2.493 *P* = 0.002)and diabetes duration(OR = 1.250 95% CI: 1.064–1.496 *P* = 0.007)were also statistically significant in the multivariate analysis.

**Table 2:** The correlation between stages of DR and the following lipid profiles

Variables	Spearman Correlation Analysis		Partial Correlation Analysis	
	r	*P*-value	r	***P***-value
RC (mmol/L)	0.360	0.000**	–	–
TC (mmol/L)	0.142	0.002*	0.289^a^	0.000**
TG (mmol/L)	0.068	0.148	0.324^b^	0.000**
LDL-C (mmol/L)	−0.016	0.736	0.332^c^	0.000**
HDL-C (mmol/L)	0.054	0.254	0.327^d^	0.000**
APOA (g/L)	0.003	0.947	0.334^e^	0.000**
APOB (g/L)	0.066	0.165	0.329^f^	0.000**

Abbreviations: *APOA* apolipoprotein A, *APOB* apolipoprotein B, *RC* Remnant cholesterol, *TC* total cholesterol, *TG* triglycerides, *LDL-C* low-density lipoprotein cholesterol, *HDL-C* high-density lipoprotein cholesterol, *APOA* apolipoprotein A, *APOB* apolipoprotein B

Notes: associations between serum lipid profile and stages of DR by Spearman correlation analysis and the association between RC and stages of DR by partial correlation analysis ^a^Adjusted for TC; ^b^Adjusted for TG; ^c^Adjusted for LDL-C; ^d^Adjusted for HDL; ^e^Adjusted for APOA; ^f^Adjusted for APOB. *P < 0.05, **P < 0. 001

**Table 3:** The association between RC value and DR

Factors	Univariate model			Multivariate model		
	OR	95%CI	*P*-value	OR	95%CI	***P***-value
Age, per10y increase	1.221	1.051–1.418	0.009**	1.002	0.981–1.022	0.880
Diabetes duration, per 1y increase	1.357	1.184–1.555	0.000**	1.072	1.036–1.110	0.000^**^
DPN, yes or no	1.561	1.067–2.284	0.022	1.201	0.770–1.873	0.419
Anitihypertensive drugs, yes or no	0.6	0.458–0.994	0.046	1.062	0.666–1.690	0.800
SBP, per10mmHg increase	1.16	1.043–1.289	0.006*	1.004	0.992–1.018	0.498
FBG, per 1 mmol/L increase	1.04	0.976–1.108	0.228			
HbA1c per1% increase	1.078	0.971–1.197	0.159			
Uric acid per 1 μmol/l increase	1.00	0.998–1.001	0.61			
TC, per 1 mmol/L increase	1.367	1.145–1.632	0.000**	0.936	0.731–1.198	0.598
TG, per 1 mmol/L increase	1.087	0.995–1.187	0.064			
HDL-C, per 1 mmol/L increase	1.315	0.676–2.556	0.42			
LDL-C, per 1 mmol/L increase	1.007	0.793–1.279	0.956			
eGFR, per10^-1^^-1^ (ml/min/1.73m^2^) increase	0.93	0.844–1.026	0.147			
mAlb > 30 mg/24 h, yes or no	1.003	1.001–1.005	0.003*	1.334	0.814–2.185	0.252
TyG-index per1 increase	1.393	1.095–1.772	0.007	0.766	0.554–1.059	0.106
RC, per 1 mmol/L increase	6.823	4.011–11.609	0.000**	5.623	2.996–10.556	0.000**
NLR per1 increase	1.801	1.310–2.477	0.000**	1.598	1.121–2.279	0.01*

Abbreviations: *DR* diabetic retinopathy, *DPN* diabetic peripheral neuropathy, *BMI* body mass index, *SBP* systolic blood pressure, *FBG* Fasting blood glucose, *HbA1c* glycosylated hemoglobin, *TC* total cholesterol, *TG* triglyceride, *HDL-C* high-density lipoprotein cholesterol, *LDL-C* low-density lipoprotein cholesterol, *APOA* apolipoprotein A, *APOB* apolipoprotein B, *eGFR* estimated glomerular filtration rate, *TyG-index* triglyceride glucose index, *SBP* systolic blood pressure, *UA* Uric acid, *NLR* Neutrophil–Lymphocyte ratio, *TyG-index* triglyceride glucose index, *mAlb* microalbuminuria

Notes: Binary Logistic Regression Analysis.*P < 0.05, **P < 0. 001

### RC, NLR, and diabetes duration predicted value of DR

**Fig. 1:**
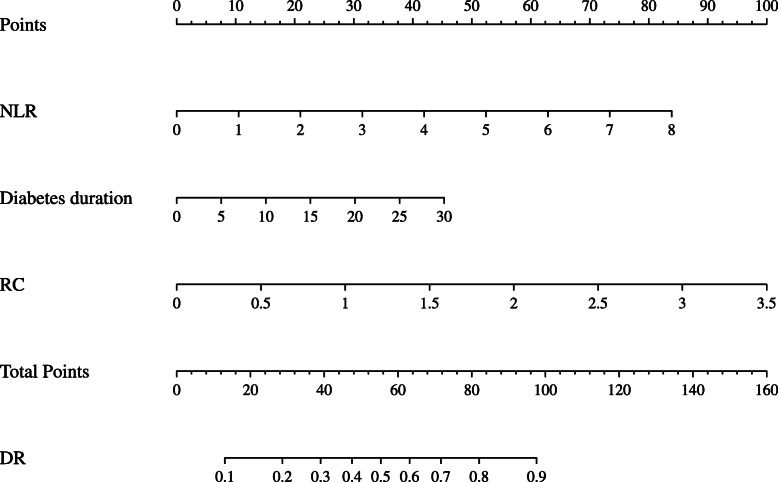
Nomogram showed the risk of DR. Abbreviations: DR: diabetic retinopathy; RC: remnant cholesterol; NLR: neutrophil-lymphocyte ratio

The multivariate regression analyses derived the nomogram of DR prediction in Fig. 1, composed of RC, NLR, and diabetes duration. The ROC analysis highlights the data scope, with the range of the ROC curve (AUC) for RC, NLR, diabetes duration, and the nomogram of 0.714 (95%CI 0.672–0.755; *p* < 0.001), 0.597 (95%CI 0.545–0.649; *P* < 0.001), 0.672 (95%CI 0.623–0.721; *P* < 0.001), and 0.758 (95%CI 0.714–0.802 *P* < 0.001), independently (Fig. S1).

### The relationship between RC level and DR, every stage of DR and DME

**Fig. 2:**
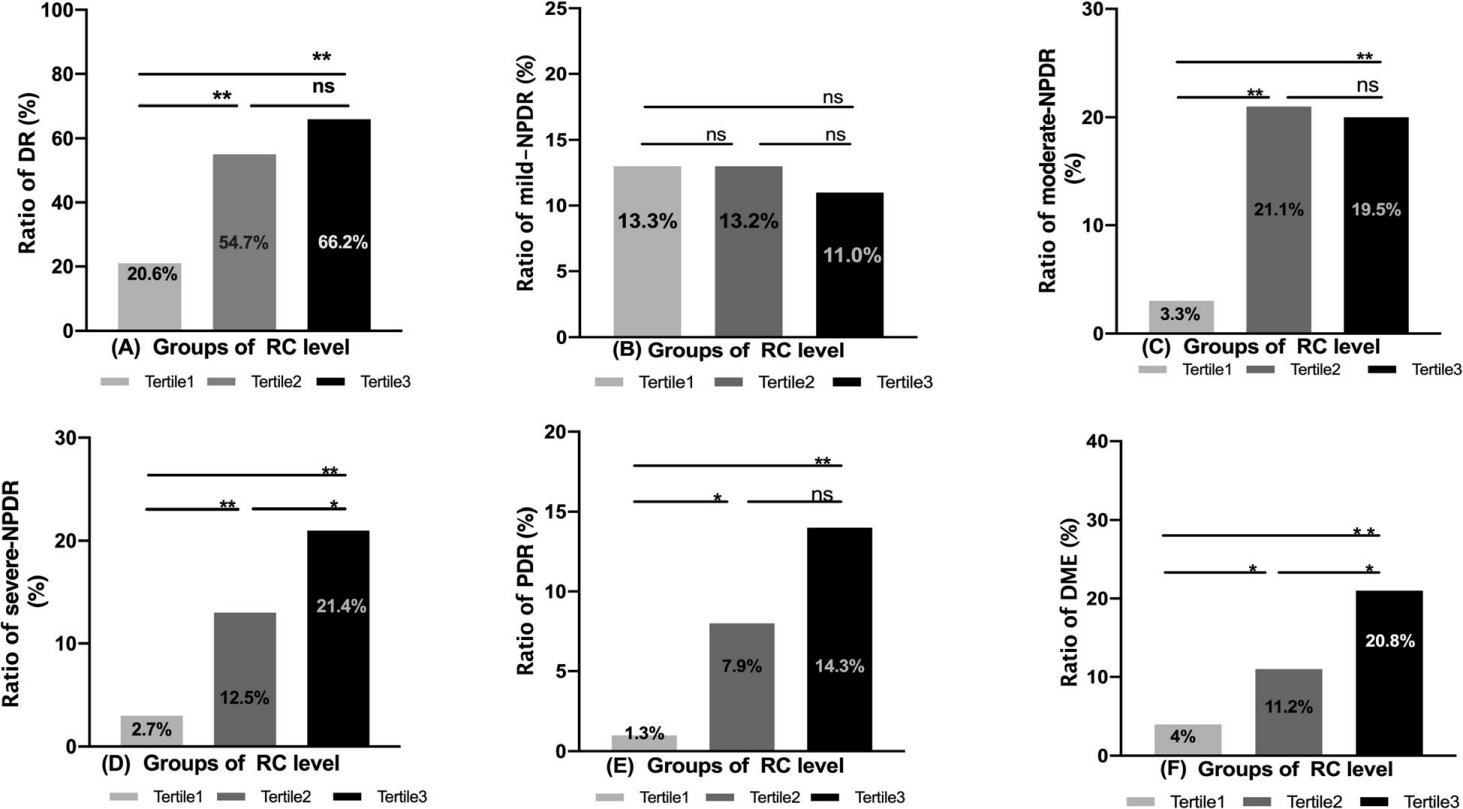
The relationship between RC levels and DR, stages of DR, and DME. Abbreviations: DR: diabetic retinopathy; NPDR: non-proliferative diabetic retinopathy; PDR: proliferative diabetic retinopathy; DME: diabetic macular edema. Notes: The RC level was divided into three groups by tertile, Tertile1 group (< 0.52 mmol/l, *N* = 150), Tertile2 group (0.52–0.83mmo/l, *N* = 152) and Tertile3 group (**>** 0.83 mmol/l, *N* = 154). Chi-square test was used for comparison between groups, *P < 0.05, **P < 0. 01

The RC levels were divided into three teams as per tertile; Tertile1 team (< 0.52 mmol/, *N* = 150), Tertile 2 team (0.52–0.83mmo/l, *N* = 152) and Tertile3 group (**>** 0.83 mmol/l, *N* = 154). In contrast with the Tertile 1 team, the ratio of DR, moderate-NPDR, severe-NPDR, PDR, and DME in the Tertile3 group increased significantly (all *P* < 0.01), but the ratio of mild-NPDR yielded no statistically significant difference (Fig. 2).

### Relationship between RC and other potential risk factors of DR

The correlation between RC and other potential risk factors of DR was assessed using spearman correlation analysis, with the results suggesting that BMI (*r* = 0.104), HbA1c (*r* = 0.101), TC (*r* = 0.559), TG (*r* = 0.477), LDL-C (*r* = 0.136), APOB (*r* = 0.398), UA (*r* = 0.15), mAlb (*r* = 0.179) and TyG-index (*r* = 0.488) were positively correlated with RC (all *P* < 0.05, Table 4).

**Table 4:** The correlation between RC value and other potential risk factors of DR

Variables	Spearman Correlation Analysis	
	r	***P***-value
Age (years)	−0.007	0.884
Diabetes duration (years)	0.068	0.149
BMI (kg/m^2^)	0.104	0.027*
Fasting C-pepide (ng/ml)	0.007	0.885
HbA1c (%)	0.101	0.031*
TC (mmol/L)	0.559	0.000**
TG (mmol/L)	0.447	0.000**
HDL-C (mmol/L)	0.085	0.069
LDL-C (mmol/L)	0.136	0.002*
APOA (g/L)	0.033	0.49
APOB (g/L)	0.398	0.000*
eGFR (ml/min/1.73m^2^)	−0.004	0.927
UA (μmol/L)	0.15	0.000**
mAlb (mg/24 h)	0.179	0.000**
NLR	0.035	0.463
TyG-index	0.488	0.000**

Abbreviations: *DR* diabetic retinopathy, *BMI* body mass index, *HbA1c* glycated hemoglobin, *TC* total cholesterol, *TG* triglyceride, *HDL* high-density lipoprotein, *LDL* low-density lipoprotein, *APOA* apolipoprotein A, *APOB* apolipoprotein B, *eGFR* estimated glomerular filtration rate, *TyG-index* triglyceride glucose index, *UA* Uric Acid, *NLR* Neutrophil–Lymphocyte ratio, *TyG-index* triglyceride glucose index, *mAlb* microalbuminuria

Notes: Spearman Correlation Analysis. *P < 0.05, **P < 0. 001

## Discussion

The current research findings offer novel evidence that the circulating level of RC is positively correlated with the occurrence and severity of DR (Table 3, Fig. 3). RC may be another risk factor for DR, in addition, to complete cholesterol, low-density lipoprotein, triglycerides, and traditional blood lipid profiles. However, the contribution of high-level RC in mild DR was minor, and the damage of RC to microvascular environments in diabetic patients may be a chronic and long-term process [17].

It is well known that most T2DM patients have relatively high BMIs, insulin resistance, and insulin secretion disorders [18], increasing the number of cholesterol-rich VLDL residual particles [19]. TyG-index is a proxy for insulin resistance and is highly correlated with the gold standard hyperinsulinemia-orthoglycemic clamp [20], which can be a good assessment of insulin resistance in patients. It has been reported that RC was correlated with insulin resistance [21]. In the current study, TyG-index is associated with RC (r = 0.488 *P* < 0.001). Insulin resistance leads to elevated RC levels, which may be involved in the occurrence of DR [22].

It is widely believed that NLR can be used as an inflammatory marker for some non-infectious diseases [23, 24]. In this study, there is a significant difference between the DR group and the non-DR team [1.6 (1.3–1.93) vs1.69 (1.41–2.76) *P* < 0.01], which was consistent with the results of Wang et al. [25]. In addition, a low-grade inflammatory response is one of the recognised pathogenesis of DR. It is associated with oxidative stress, high expression of inflammatory factors and the formation of advanced glycation end products (AGEs). Notably, RC can also trigger an inflammatory response, causing damage to the vascular endothelium [8]. Animal experiments have confirmed that RC particles easily enter the intima of the artery due to their particles size and composition characteristics, do not readily diffuse back into the blood, and can be directly ingested by macrophages [26]. Macrophages promote lipid decomposition, and triglyceride-rich lipoproteins (TGRLs) generate core components, especially high levels of fatty acids and saturated fatty acids, which combine with toll-like receptors 4 and 2, induce inflammasome activation, promote interleukin-1β secretion, and amplify inflammatory effects [27]. Varbo’s clinical study on the relationship between RC and LDL and ischemic heart disease and low-grade systemic inflammation reported that only elevated RC levels had a causal relationship with low-grade systemic inflammation [28]. In the current study, RC and NLR were higher in the DR group, but there was no apparent correlation. The relationship between the two needs to be further validated by large sample size studies.

Additionally, there was a dynamic relationship between RC and UA (r = 0.15 *P* < 0.001). Some studies have reported that a high standard of UA is closely associated with oxidative stress and inflammation [29]. Other studies suggested that UA can protect vascular endothelium, which may be caused by the antioxidant effect of UA concentrations in a particular range [30]. However, inflammatory responses can be stimulated [31]. Notably, a sizeable cross-sectional study of a rural Chinese populace reported that high uric acid levels might be associated with various blood lipids [32]. Do increased UA levels contribute to the RC reduction? Investigating the relationship between the two is worthy of further research.

RC may be a risk factor for both macrovascular and microvascular diseases in diabetes patients. A large cohort study found that residual cholesterol, rather than LDL-C, may be the primary cause of arteriosclerotic cardiovascular disease. The relative risk of major adverse cardiovascular events increased by 21% for every 10 mg/dL increase in residual cholesterol levels (about 0.26 mmol/L) [10], and a second cross-sectional study in China showed that RC was related to the risk of cardiovascular death in T2DM patients with newly diagnosed stage 3–5 DN [33]. Furthermore, a recent cohort study suggests that RC is associated with vision-threatening DR in type 1 diabetes [34]. The present study selected type 2 diabetic patients without coronary heart disease to avoid patients affected by the relationship between RC and diabetic retinopathy. Finally, in this study, microalbuminuria was related to RC (r = 0.179 *P* < 0.001), but the relationship between RC and DR was not disturbed after adjusting for the 24-h microalbumin variable using binary logistic regression (Tables 4 and S2).

The two primary visual-threatening complications of diabetic retinopathy are the proliferative phase of retinal neovascularisation and macular oedema, leading to the destruction of the central blood-retinal obstacle. In this study, the incidence of PDR and DME increased significantly in the high RC level group (Fig. 2). Related meta-analysis and cross-sectional studies suggest that the occurrence of DME is closely associated with high triglyceride levels [35], and the current study found that RC correlates with TG (r = 0.447 *P* < 0.001). RC may be a risk factor masked by TG for DME and PDR. There is evidence of considerable residual risk of cardiovascular events even after LDL-C reduction or HDL-C increase to recommended concentrations with statins and other lipid-lowering agents due, in part, to elevated RC levels [36]. Similarly, the elevation of RC levels is important for preventing and delaying DME and PDR.

The nomogram can predict the risk of disease occurrence and has attracted more attention due to its simple, intuitive, and reference value. Therefore, RC, NLR, and diabetes duration were selected and contributed to the most stable model in multivariate logistic regression analysis and resulted in the predicted risk of DR nomogram. Diagnostic efficiency was 0.758 (95%CI 0.714–0.802 *P* < 0.001). For communities or hospitals without facilities for fundus examination, physicians can use this predictive model to screen people at higher risk of DR.

Although the relationship between HbA1c and DR is widely recognised [37, 38], the current study found no significant difference in HbA1c between the DR group and the non-DR participants [8.5 (7.6–9.8) vs 8.2 (7.1–9.5) *P* = 0.076]. Those participants that were hospital inpatients often had poorly controlled glycemic levels, which may have caused this discrepancy. Interestingly, a similar phenomenon was also reported in other studies [39, 40]. It should be acknowledged that selection bias is challenging to avoid when hospital inpatients are participants in research studies.

The effects of RC on patients with type 2 diabetes may involve the imbalance of oxidative and antioxidant capacity [27], RC is involved in the damage of the vascular endothelium [8] and the activation of the inflammatory response. Also, insulin resistance may be involved in the increase of RC [21]. These effects have been confirmed in previous studies on macrovascular complications in T2DM patients. However, the correlation between RC and DR was confirmed in this study based on the hospitalisation of type 2 diabetes mellitus for the first time.

### Study advantages and limitations

The advantage is that patients with type 2 diabetes with cardiovascular disease were excluded in this study in order to further explore the correlation between the occurrence and severity of RC and DR. However, also some limitations in this research. For example, the study was undertaken in a single-centre, with the observational design based on a relatively cross-sectional analysis with an insufficient sample size. Notably, the prevalence of dyslipidemia and other risk elements in the Asian population was similar to other extensive contemporary tests and real-world registries involving ethnic groups [41, 42], which may support the generality of the study outcomes. Secondly, RC is correlated with DR, but the causal relationship between RC and DR cannot be proven. Thirdly, to exclude the influence of lipid-lowering drugs on lipid profile metabolism, subjects in this study had not taken any lipid-lowering drugs orally in recent three months. This study cannot differentiate whether lipid-lowering oral medicines impact RC levels. Besides, the DR risk nomogram only has internal validation, and its diagnostic efficacy may be reduced without an external warranty.

## Conclusions

In patients with T2DM, high RC levels positively correlate with the occurrence and severity of DR. These striking findings suggested that DR may not only be affected by high blood glucose levels but also influenced by a complex lipid profile. Clinicians and patients could easily predict the occurrence of DR through a nomogram considering RC, NLR, and diabetes duration. Insulin resistance interventions may help reduce RC level and control DR risk, which must be investigated in future multicenter or community-based prospective cohort studies.

## Supplementary Information

**Additional file 1: Table S1** The association between RC value and DR. **Table S2** Effects of other variables on the relationship between RC and DR.

**Additional file 2: Fig. S1** ROC Curve for RC, NLR, DM Duration and Risk of DR Nomogram.

## Data Availability

The authors declare the data related to this study can be available from the corresponding author upon reasonable request.

## Abbreviations

DR: Diabetic retinopathy
DPN: Diabetic peripheral neuropathy
BMI: Body mass index
SBP: Systolic blood pressure
DBP: Diastolic blood pressure
FBG: Fasting blood glucose
APOA: Apolipoprotein A
APOB: Apolipoprotein B
TC: Total cholesterol
TG: Triglyceride
HDL-C: High-density lipoprotein cholesterol
LDL-C: Low-density lipoprotein cholesterol
RC: Remnant cholesterol
eGFR: Estimated glomerular filtration rate
HbA1c: Glycosylated hemoglobin
TyG-index: Triglyceride glucose index
NLR: Neutrophil to lymphocyte ratio
DPP-IV: Dipeptidyl peptidase-IV
mAlb: Microalbuminuria
ROC: Receiver operating characteristic
AUC: Area under the curve
DME: Diabetic macular oedema
