# Supplementary material for: High remnant cholesterol level is relevant to diabetic retinopathy in type 2 diabetes mellitus
Source: Lipids Health Dis. 2022 Jan 20;21:12. doi: 10.1186/s12944-021-01621-7 (PMC8772129; doi:10.1186/s12944-021-01621-7)
Supplement: Supplementary file 1 — Additional file 1: Table S1 The association between RC value and DR. Table S2 Effects of other variables on the relationship between RC and DR. [file 12944_2021_1621_MOESM1_ESM.docx]

**Supplementary Material**

**Table S1** The association between RC value and DR

|  | **OR** | **95%CI** | *P***-value** |
| --- | --- | --- | --- |
| Model 1 | 4.224 | 2.657-6.716 | 0.000** |
| Model 2 | 4.238 | 2.666-6.736 | 0.000** |
| Model 3 | 3.952 | 1.262-12.376 | 0.018* |

Abbreviations: DR: diabetic retinopathy; DPN: diabetic peripheral neuropathy; HbA1c: glycated hemoglobin; TC: total cholesterol; TG: triglyceride; LDL-C: low-density lipoprotein; eGFR: estimated glomerular filtration rate; TyG-index: triglyceride glucose index; NLR: Neutrophil–Lymphocyte ratio; TyG-index: triglyceride glucose index; mAlb: microalbuminuria.

Notes: Binary regression analysis; Model 1: adjusted for age, sex, and diabetes duration; Model2: Model1 + adjusted for smoking yes or not, hypertension yes or not, Insulin use yes or not, and antihypertensive drugs use yes or not; Model 3: Model 2+ adjusted for SBP, BMI, TC, TG, LDL-C, eGFR, HbA1c, NLR, TyG, mAlb>30mg/24h yes or not, and DPN yes or not. **P*<0.05, ***P*<0. 001.

**Table S2** Effects of other variables on the relationship between RC and DR

| **Variables** | **N** | **OR** | **95%CI** | *P***-value** |
| --- | --- | --- | --- | --- |
| **HbA1c (%)** |  |  |  |  |
| <7 | 88 | 3.695 | 1.38-9.897 | 0.007^*^ |
| ≥7 | 368 | 4.077 | 2.482-6.697 | 0.000^**^ |
| **mAlb (**mg/24h**)** |  |  |  |  |
| <30 | 367 | 3.364 | 2.038-5.553 | 0.000^**^ |
| ≥30 | 89 | 5.801 | 2.251-14.95 | 0.000^**^ |

Abbreviations: DR: diabetic retinopathy; mAlb: microalbuminuria; HbA1c: glycated hemoglobin;

Notes: Binary regression analysis, **P*<0.05, ***P*<0. 001.

**Figure S1 RC, NLR, and diabetes duration predicted value of DR**

Abbreviation: ROC, receiver operating characteristic; AUC, area under the curve; DR, diabetic retinopathy; NLR, neutrophil–Lymphocyte ratio; RC, remnant cholesterol; DM duration, diabetes mellitus duration.
