# Supplementary figures and images for: High remnant cholesterol level is relevant to diabetic retinopathy in type 2 diabetes mellitus
Source: Lipids Health Dis. 2022 Jan 20;21:12. doi: 10.1186/s12944-021-01621-7 (PMC8772129; doi:10.1186/s12944-021-01621-7)

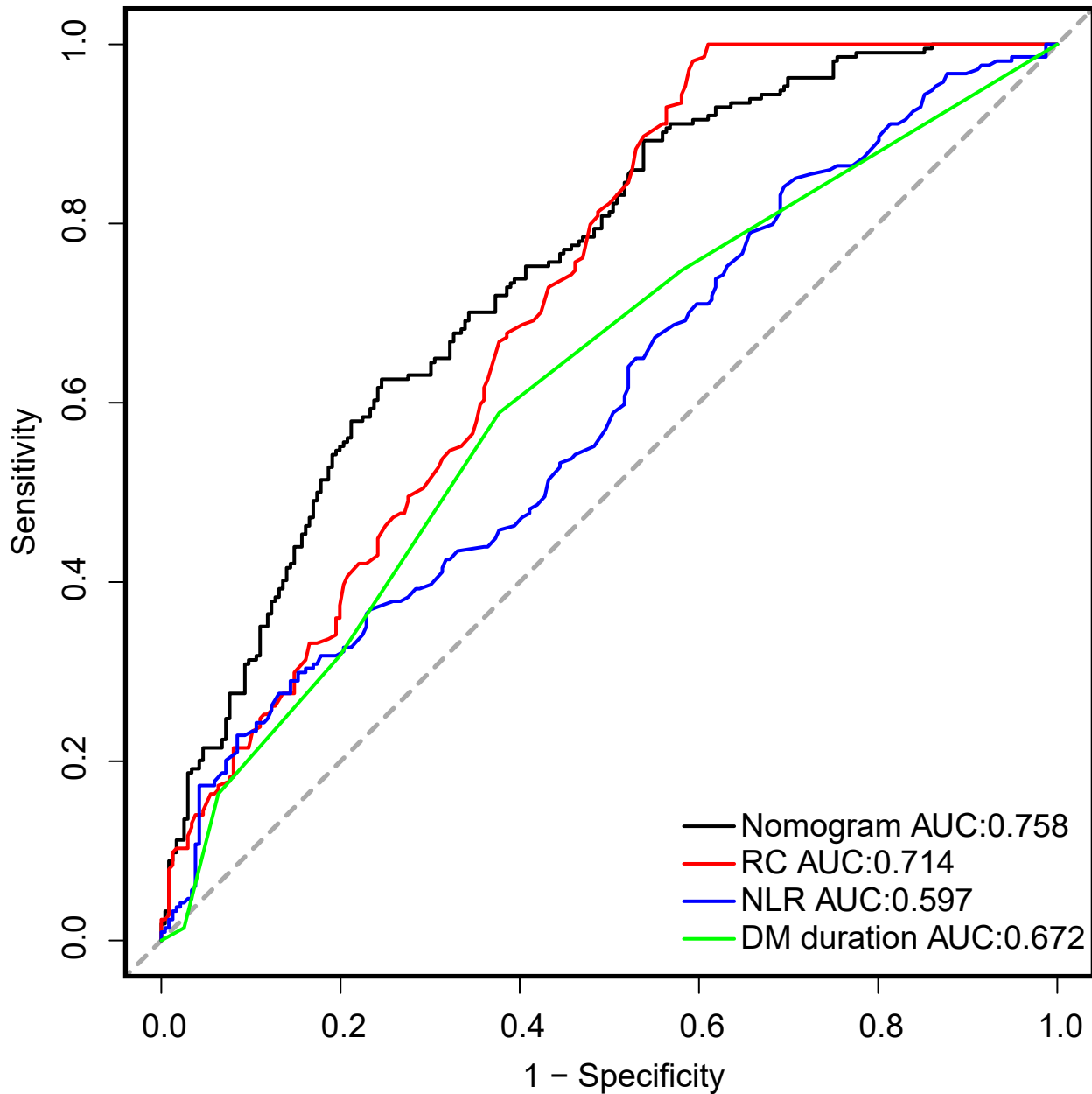

Supplement: Supplementary file 2 — Additional file 2: Fig. S1 ROC Curve for RC, NLR, DM Duration and Risk of DR Nomogram. [file 12944_2021_1621_MOESM2_ESM.pdf]
